# Effect of aloe vera juice on type 2 diabetes mellitus among Indian patients

**DOI:** 10.6026/973206300191015

**Published:** 2023-10-31

**Authors:** Durairaj Prakash, John Margaret, N. Siva Subramanian, Chaudhari Mishva Maheshbhai, Chaudhari Keyaben Babubhai, Chaudhari Sanjanaben Kiranbhai, Seemaben Kanubhai Chaudhary, Chaudhary Urvashiben Popatbhai, Hardik Lokesh Kumar Lodha

**Affiliations:** 1 Nootan College of Nursing, Sankalchand Patel University, Visnagar, Gujarat-384315,India

**Keywords:** Aloe vera juice, Type 2 diabetes mellitus, Blood glucose, Patients

## Abstract

It is of interest to assess the effect of Aloe vera juice on type 2 diabetes mellitus. Non-probability convenience sampling techniques was used to obtain sample of fifty type 2 diabetic patients who satisfied the inclusion criteria. Blood sugar level was assessed by using glucometerbefore and after consumption of Aloe vera juice. The average post-test score in the experimental group 177.43 (Standard deviation 17.64) was significantly lower than the average post-test score in control group 128.76 (Standard deviation 27.50). Unpaired 't' value 7.2926. It shows there is a significant difference found in the post-test scores on the level of blood glucose. Chi square analysis showed that there was no correlation between patients with demographic variables except age, occupation, smoking habit, habit of alcoholism and family history.

## Background:

**Figure 1:**
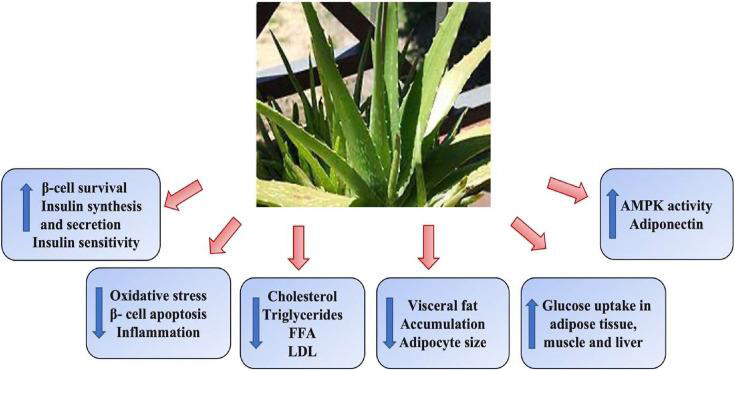
Benefits of Aloe vera

Hyperglycemia caused by diabetes mellitus can be brought on by either insulin resistance, inadequate insulin production, or both [1]. Type 1, type 2, and gestational diabetes mellitus are the three different types of diabetes mellitus; type 2 diabetes mellitus is the most prevalent kind (90-95%) [2]. Because of its rising prevalence and effects, diabetes mellitus poses a threat to world health [3]. According to estimates, there were 463 million diabetics worldwide in 2019 and that number was expected to rise to 578 million in 2030 and 700 million in 2045 [4].Diabetes medications and medical devices largely contribute to the significant financial expense that the disease inflicts on affected individuals and society [5]. Long-term usage of diabetes medications has also been linked to kidney damage and cancer [6]. On the other hand, diabetes and pre diabetes must be treated and prevented in order to enhance wellbeing and lower expenses [7]. As an alternative therapy, food based therapy is advised since it is more maintainable and less prone to have adverse effects [8]. The Liliaceae family, which comprises over 200 species worldwide, includes the succulent plant Aloe. The most popular species of Aloe, Aloebarbadensis, is also known as Aloe vera. Aloe vera has been utilised in health and cosmetic products for thousands of years because of its laxative, anti-inflammatory, and anti-tumor properties (Figure 1). It includes over 75 active substances, the bulk of which seem to be biologically significant in the treatment of disease. These substances include enzymes, vitamins, carbohydrates, minerals, lignin, amino acids, and salicylic acid. The medicinal plant Aloe vera has been utilised for centuries as an anti hyperglycemic drug that may be helpful in the treatment of diabetes and pre diabetes [9, 10]. It has been noted to improve insulin sensitivity and blood glucose regulation [11]. Numerous chemical elements, including chromium and alprogen, which are prevalent in Aloe vera, can repair injured pancreatic beta cells, increase insulin action, and lower blood sugar levels [12]. Figure 1 shows various health benefits of Aloe vera juice, this including insulin synthesis, anti-oxidants, cholesterol control and smoothening of the skin. Therefore, it is of interest to assess the effect of Aloe vera juice on type 2 diabetes mellitus.

## Methodology:

The impact of aloe vera juice on type 2 diabetes mellitus was evaluated using a quasi-experimental research method. Using a non-probability convenience sampling technique, 60 diabetic patients who met the inclusion criteria were gathered. They were picked at random following verbal research information and their consent. Demographic data, including age, education, sex, religion, occupation, income, dietary habits and family history were gathered using a structured interview schedule. The blood glucose level was measured using glucometer, level of blood glucose divided into four categories normal, stage I, II, III, IV (Table 1). Following evaluation, 20 ml of Aloe vera juice was given to the experimental group with instructions to consume it every day for 30 days. A daily record sheet was included for keeping track of consumption. The prescribed anti-diabetic medication was to be continued for the control group. The researcher calculated the analysis from the data using descriptive and inferential statistics manually. According to Table 1, the normal blood glucose range is between 70 and 125 mg, stage I is between 126 and 150 mg, stage II is between 151 and 175 mg, and stage III is between 176 and 200 mg. Table 2 shows Aloe vera extract specification; this contains appearance, solubility, taste, PH, odor and other microbiological analysis.

## Results:

Polite and Hungler (1999) described analysis as "a process of organizing and synthesizing data in such a way that research question can be answered and hypothesis tested. Interpretation is refers to process of making sense of the results and of examining the implication of the finding within a broader context.

**Figure 2:**
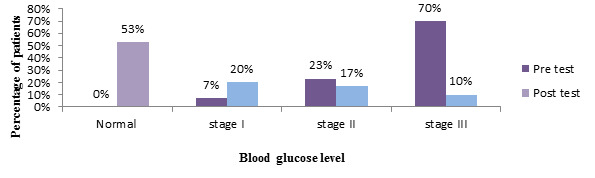
Frequency and percentage distribution of blood glucose in experimental group

Figure 2 shows frequency and percentage distribution of blood glucose in experimental group depicts that, in the pretest majority 21(70%) people were in the stage 3 diabetes mellitus, 7(23%) were in stage 2 diabetes mellitus , 2(7%) were in stage 1 diabetes mellitus. Whereas in post-test majority 16(53%) were in normal, 6(20%) were in stage 1 diabetes mellitus and 5(17%) were in stage 2 diabetes mellitus and 3(10%) were in stage 3 diabetes mellitus.

**Figure 3:**
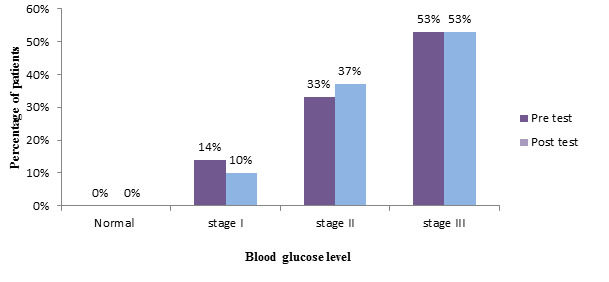
Frequency and percentage distribution of blood glucose in control group

Figure 3 shows frequency and percentage distribution of blood glucose in control group depicts that, in the pretest majority 16(53%) of people were in the Stage 3 diabetes mellitus, 10(33%) were in stage 2 diabetes mellitus,4(14%) were in stage 1 diabetes mellitus . Whereas in post-test 16(53%) of people were in the Stage 3 diabetes mellitus, 11(37%) were in stage 2 diabetes mellitus, 3(10%) were in stage 1 diabetes mellitus. Table 3 shows that the average pre-test was 177.43 (Standard Deviation 17.64) and the post-test average score is 128.76 (Standard Deviation 27.50). The paired 't' value was 13.15, this shows that there is significant difference between pre-test and post-test scores on the level of blood glucose among people with type 2 diabetes mellitus. It shows that Aloe vera juice consumption has effective in reduction of blood glucose level among people with type 2 diabetes mellitus. In the control group mean was 170.96 (Standard Deviation 18.34) and the post-test mean score is 172.26 (Standard Deviation 17.64), the paired 't' value was 7.2926. This shows that aloe vera juice was more effective in reduction of blood glucose level in type 2 diabetic patients. Table 4 shows that there was no association between post-test level of blood glucose with selected demographic variable except age, education, occupation and use of medication. Table 5 shows that there was no association between post-test level of blood glucose with selected demographic variable except age, occupation, smoking habit, habit of alcoholism and family history.

## Discussion:

The study's objective was to assess how Aloe vera juice affected type 2 diabetes mellitus. According to the study's findings, patients with type 2 diabetes who were in the experimental group showed a substantial difference in their post-test blood glucose scores after consuming Aloe vera juice. The results of the Monika C research study in Punjab, which provided support for our current investigation, revealed that diabetic patients' blood glucose, lipid profile, and blood pressure all significantly decreased after consuming aloe vera [13].Another study by Zhang Y found that Aloe vera could successfully lower FBG, HbA1c, triglyceride, TC, and LDL-C levels on pre-diabetes and early untreated diabetic patients while increasing HDL-C levels [14].These conclusions are supported by the current investigation. A study conducted by Felipe Araya Q revealed that moderate to high quality of evidence in favor of the effects of aloe vera in patients with T2DM and pre-diabetes. [15]. Neha conducted a clinical study, and according to her, Aloe vera and its constituents have been used in traditional medicine for a long time for a variety of biological activities, including hypoglycaemic, antioxidant, ant carcinogenic, anti-inflammatory, and wound healing effects through various mechanisms. These biological activities have been well covered in literature. There aren't many studies on Aloe Vera's capacity to treat diabetic dyslipidemia, though. She concentrated on the potential role of Aloe vera and its active ingredients in treating diabetic dyslipidemia as well as their mechanisms of action in pre-clinical and clinical investigations in this systematic review [16]. According to a study by Bunyapraphatsara, numerous alternative therapies are highly helpful in the treatment of diabetes. He used aloe Vera juice, which had an impact on lowering blood glucose levels. One of the most traditional and popular natural supplements for lowering blood sugar is cinnamon. Cinnamon functions as an insulin sensitizer, essentially assisting insulin in managing blood glucose [17]. Aloe vera was found to be more effective at lowering blood glucose levels among Diabetes Mellitus patients according to a study by Fatemeh [18]. According to Alinejad using Aloe vera extract in pre-diabetic individuals can reverse reduced blood sugar levels in four weeks [19]. Aloe vera has exceptional healing properties. Aloe vera lowers blood glucose levels in diabetics. Additionally, it increases the sensitivity of body tissues to insulin, increasing the efficiency of insulin. The active ingredients in aloe vera also help lower blood pressure.

## Conclusion:

Data shows that aloe vera is more effective than other treatments for type 2 diabetes.

## Figures and Tables

**Table 1:** Diabetes mellitus category

**Level of blood glucose**	**Interpretation**	**Score**
70-125 mg	Normal	0
126-150 mg	Stage I	1
151-175 mg	Stage II	2
176-200 mg	Stage III	3

**Table 2:** Aloe vera extracts specification

**Test parameter**	**Speciﬁcation**	**Result**
Appearance	white	Complies
Solubility	Soluble in water	Complies
Taste	Slightly bitter	Complies
pH	4.0-7.5	6.2
Odor	Aloe vera odor	Complies
Assay of a glycons as Aloe-emodin	NLT 50%	51.85%
Heavy metals as per USP	NMT 5 ppm	Complies
Particle size	100% passes through 20 mesh	Complies
Content of polysaccharides	NLT 40%	41.32%
*Microbiological analysis (Cfu/gm)*
*(a) Escherichia coli*	Absent	Complies
*(b) Coliforms*	Absent	Complies
*(c) Salmonella*	Absent	Complies
*(d) Pseudomonas*	Absent	Complies
*(e) Staphylococcus*	Absent	Complies
*(f) Yeast*	NMT 100 Cfu/gram	30Cfu/gram
NLT = not less than; NMT = not more than; Cfu = colony-forming unit.

**Table 3:** Comparison of the pre and post-test scores of levels of blood glucose with type 2 diabetes in experimental group.

**Group**	**test**	**Mean**	**SD**	**'t' value**	**Table value**	**Inference**
Experimental group	Pre-test	177.43	17.64	13.15	2.045	Significant
	Post-test	128.76	27.5			
Control group	Pre-test	170.96	18.34	1.22	2.045	Non-significant
	Post-test	172.26	17.64			

**Table 4:** Association between post-test levels of blood glucose level with their selected demographic variables in experimental group

**S. No**	**Demographic variables**		**Frequency**				**Chi Square Value**	**Table value**	**Inference**
			**Normal**	**Stage I**	**Stage II**	**Stage III**			
1	Age	25-30	1	0	2	0			
		30-35	2	1	3	1	9.8	2.262	S
		35-40	2	6	2	2			
		Above 40	0	2	3	3			
2	Education	No-formal education	0	2	1	1			
		Primary to high school	2	3	3	3	4.2609	2.262	S
		Higher secondary school	1	2	3	2			
		Graduate	0	4	2	1			
3	Occupation	None	0	1	2	1			
		Farmer	2	7	3	2	5.993	2.262	S
		Job	2	3	1	0			
		Business	1	2	1	2			
4	Religion	Hindu	7	6	8	6			
		Muslim	0	1	1	1	1.136	2.262	NS
		Christian	0	0	0	0			
		Others	0	0	0	0			
5	Dietary habit	Vegetarian	6	7	6	8			
		Non-vegetarian	1	0	1	1	1.0732	3.182	NS
6	Smoking habit	Yes	4	3	3	4	1.5265	3.182	NS
		No	3	10	3	10			
7	Habit of alcoholism	Yes	1	1	1	1	1.296	3.182	NS
		No	3	10	3	10			
8	Family history	History of DM	5	10	3	4	0.2669	3.182	NS
		No history of DM	2	3	1	2			
9	Use of medication	Yes	3	10	8	5	9.125	3.182	S
		No	3	1	0	0			
S-Significant NS-Non significant

**Table 5:** Association between post-test levels of blood glucose level with their selected demographic variables in control group

**S. No**	**Demographic variables**		**Frequency**				**Chi Square Value**	**Table value**	**Inference**
			**Normal**	**Stage I**	**Stage II**	**Stage III**			
1	Age	25-30	0	1	0	1			
		30-35	1	2	2	1	3.396	2.262	S
		35-40	3	3	4	4			
		Above 40	1	2	3	2			
2	Education	No-formal education	0	1	1	1			
		Primary to high school	2	3	3	4			
		Higher secondary school	1	3	4	2	2.1236	2.262	NS
		Graduate	1	2	1	1			
3	Occupation	None	1	2	1	1			
		Farmer	2	3	5	2	2.3347	2.262	S
		Job	1	3	2	1			
		Business	1	1	2	2			
4	Religion	Hindu	7	5	8	7			
		Muslim	0	1	1	1	1.1403	2.262	NS
		Christian	0	0	0	0			
		Others	0	0	0	0			
5	Dietary habit	Vegetarian	7	10	6	4			
		Non-vegetarian	0	2	0	1	2.593	3.182	NS
6	Smoking habit	Yes	0	8	2	2	11.42	3.182	S
		No	6	2	5	5			
7	Habit of alcoholism	Yes	0	1	4	1	4.3812	3.182	S
		No	10	4	7	3			
8	Family history	History of DM	3	5	6	4			
		No history of DM	7	1	2	2	8.707	3.182	S
9	Use of medication	Yes	5	10	7	3	1.424	3.182	NS
		No	0	2	2	1			
S-Significant NS-Non significant
